# CytoASP: a Cytoscape app for qualitative consistency reasoning, prediction and repair in biological networks

**DOI:** 10.1186/s12918-015-0179-6

**Published:** 2015-07-11

**Authors:** Aristotelis Kittas, Amélie Barozet, Jekaterina Sereshti, Niels Grabe, Sophia Tsoka

**Affiliations:** Department of Informatics, King’s College London, Strand, London, WC2R 2LS UK; Department of Medical Oncology, NCT, University of Heidelberg, Im Neuenheimer Feld 267, Heidelberg, 69120 Germany

**Keywords:** Biological networks, Regulatory networks, BioASP, Qualitative modelling

## Abstract

**Background:**

Qualitative reasoning frameworks, such as the Sign Consistency Model (SCM), enable modelling regulatory networks to check whether observed behaviour can be explained or if unobserved behaviour can be predicted. The BioASP software collection offers ideal tools for such analyses. Additionally, the Cytoscape platform can offer extensive functionality and visualisation capabilities. However, specialist programming knowledge is required to use BioASP and no methods exist to integrate both of these software platforms effectively.

**Results:**

We report the implementation of CytoASP, an app that allows the use of BioASP for influence graph consistency checking, prediction and repair operations through Cytoscape. While offering inherent benefits over traditional approaches using BioASP, it provides additional advantages such as customised visualisation of predictions and repairs, as well as the ability to analyse multiple networks in parallel, exploiting multi-core architecture. We demonstrate its usage in a case study of a yeast genetic network, and highlight its capabilities in reasoning over regulatory networks.

**Conclusion:**

We have presented a user-friendly Cytoscape app for the analysis of regulatory networks using BioASP. It allows easy integration of qualitative modelling, combining the functionality of BioASP with the visualisation and processing capability in Cytoscape, and thereby greatly simplifying qualitative network modelling, promoting its use in relevant projects.

## Background

Biological networks can be modelled at different levels of abstraction. Whereas quantitative models rely on the availability of kinetic parameters, qualitative models are primarily based on the network structure, rendering them generally applicable to large-scale analyses [1]. Among qualitative reasoning platforms, the Sign Consistency Model (SCM) [2] is a framework for modelling influence graphs by confronting a network of labelled interactions with quantitative data, imposing a collection of constraints. In the context of regulatory networks, SCM can be used to check whether an observed behaviour can be explained or if unobserved behaviour can be predicted.

Given genes, proteins, or metabolites and a graph of labelled interactions among these entities, cases are identified where experimental observations are inconsistent with known regulation patterns to indicate either unreliable data or missing regulations. It is assumed that data originates from steady-state shift experiments where, given a perturbation, the difference between two steady states corresponds to protein or metabolite changes in concentration. Logical modelling may then be employed to determine viable states of molecular interactions when the network is confronted with experimental data by using the SCM framework. Further specific experiments can also be suggested to validate the system behaviour through reconciling with the regulatory conditions needed to achieve it [3].

The BioASP software collection ^1^ implements methods for modelling metabolic and gene regulatory networks [3], using logical rules with Answer Set Programming (ASP) [4]. ASP is a form of declarative programming that can address difficult, NP-hard search problems. The building blocks for ASP programs are atoms, literals, and rules. Atoms are elementary propositions (factual statements) that may be true or false, literals are atoms and their negations, rules are expressions composed of atoms and programs are finite collections of rules [5]. The consistency problem is thus encoded by a collection of rules, such that its intended models, called *answer sets*, represent solutions to the problem.

A publicly available Python script, *ingranalyze*^2^, facilitates influence graph modelling without knowledge of ASP. However, it provides no graphical user interface and specialist user input is required (i.e. file formatting, syntactic restrictions, pre/post-processing through command line etc.). A web service^3^ also exists, which provides a point-and-click interface but no visualisation of the results.

Cytoscape [6] offers an ideal platform for network analysis encompassing visualisation, layout and processing capabilities [6]. A Cytoscape 3.x app, named CytoASP, is implemented and reported here, that uses *ingranalyze* as a back-end for influence graph consistency checking, prediction and repair operations through Cytoscape. User input is provided through a graphical interface, while integration with Cytoscape provides additional functionality features for visualisation and further analysis.

## Implementation

The interaction between Cytoscape and CytoASP was implemented in Java. This part of the program starts by displaying the graphical interface where the user selects the desired options. Once the calculation is requested by the user, it executes parallel threads, one for each of the different networks. Each of these threads writes two text files, one describing the network, and another one containing the corresponding observations. It then runs a Python script that will make use of those two text files and interact with the BioASP *ingranalyze* package.

The Python script plays the intermediary role between information in the text files and the *ingranalyze* Python package. It processes raw data from the text files and passes them on (along with the options chosen by the user) to *ingranalyze*, which executes the binaries for the *clasp* and *gringo* ASP solvers. Once the predictions are made, it obtains the results and writes them to files in the chosen directory. The Java threads finally read the result files when the Python processing is over and update the visualisation in Cytoscape accordingly.

### App functionality

The main functionality of CytoASP is the analysis of sign influence graphs, which are directed graphs where vertices are the input and state variables of a system and edges express their effects on each other. An edge *i*→*j* means that the variation of *i* through time influences the level of *j*. The edges are labeled with positive and negative signs indicating activations and inhibitions respectively.

On this basis, CytoASP provides the following functionality through Cytoscape: i) checking the network for consistency, ii) identifying minimal inconsistent cores (MICs), iii) computing repair sets, and iv) predicting node variation under consistency and repair. Assuming a steady state and some form of perturbation, the difference between initial and final states is used as input. The algorithm proceeds to determine consistency and predict unknown regulation effects and repair sets. By overlaying experimental data on the network, some of the system variables are fixed, allowing checks on whether observed data agree with underlying graph interactions.

### Consistency and repair

The SCM defines the general rule to determine network consistency: “The variation of each node (which is not considered as input) must be explained by an influence received from at least one of its predecessors” [2]. A network is considered consistent if there exists a total labelling of its edges and nodes which overrides the current labelling and which is consistent with regard to the SCM rule. This can be formulated as a boolean satisfiability problem which is known to be NP-hard and may be efficiently tackled by the ASP approach [7]. A particular instance of network, with overlaid data, is consistent if its (potentially partial) labelling is consistent.

BioASP defines different rules that allow checking the consistency of a network. These rules are then handled by a grounder and then by a solver. If the network is consistent, there will be at least one answer set. In this case, predictions may be calculated, which are the set of values for the unobserved nodes in the intersection of all possible solutions (i.e. answer sets of predicted node variations).

If the network is inconsistent, various repair options may be possible. A repair set is a set of modifications of your data or graph which makes it consistent. One is usually interested in “minimal repairs”, i.e. ones that result in the smallest number of changes in the dataset. By using BioASP, it is possible to identify all possible repair sets for the analysed network, which may be repaired using either of these options:

1. Flipping observations, i.e. changing the signs for the variation of each node.
2. Defining certain nodes as input, namely nodes which are not influenced by their predecessors.
3. Flipping influences, i.e. changing the signs of the network edges.
4. Adding new influences in the network.

Each repair set is output as a different file. As it is possible to have many repair sets, these cannot be visualised simultaneously. However, it maybe the case that some repairs are common in all repair sets, and these are visualised on the network as nodes with alternating fill and border colour (see e.g. Fig 2), or edges with alternating shape and colour. Predictions can also be made under different repair modes, which are the deductions for the variation of unobserved nodes that hold true under all repair sets.

When calculating repairs, it is often useful to provide concise explanations when an experimental profile is inconsistent with an influence graph, by isolating parts of the network responsible for the inconsistency. These are minimal sub-graphs of an influence graph with a given partial labelling, such that the vertices and edges cannot be labelled consistently. These sub-graphs are called Minimal Inconsistent Cores (MICS)s [7], and CytoASP can calculate them, by checking the appropriate setting in its options.

### Usage

**Fig. 1:**
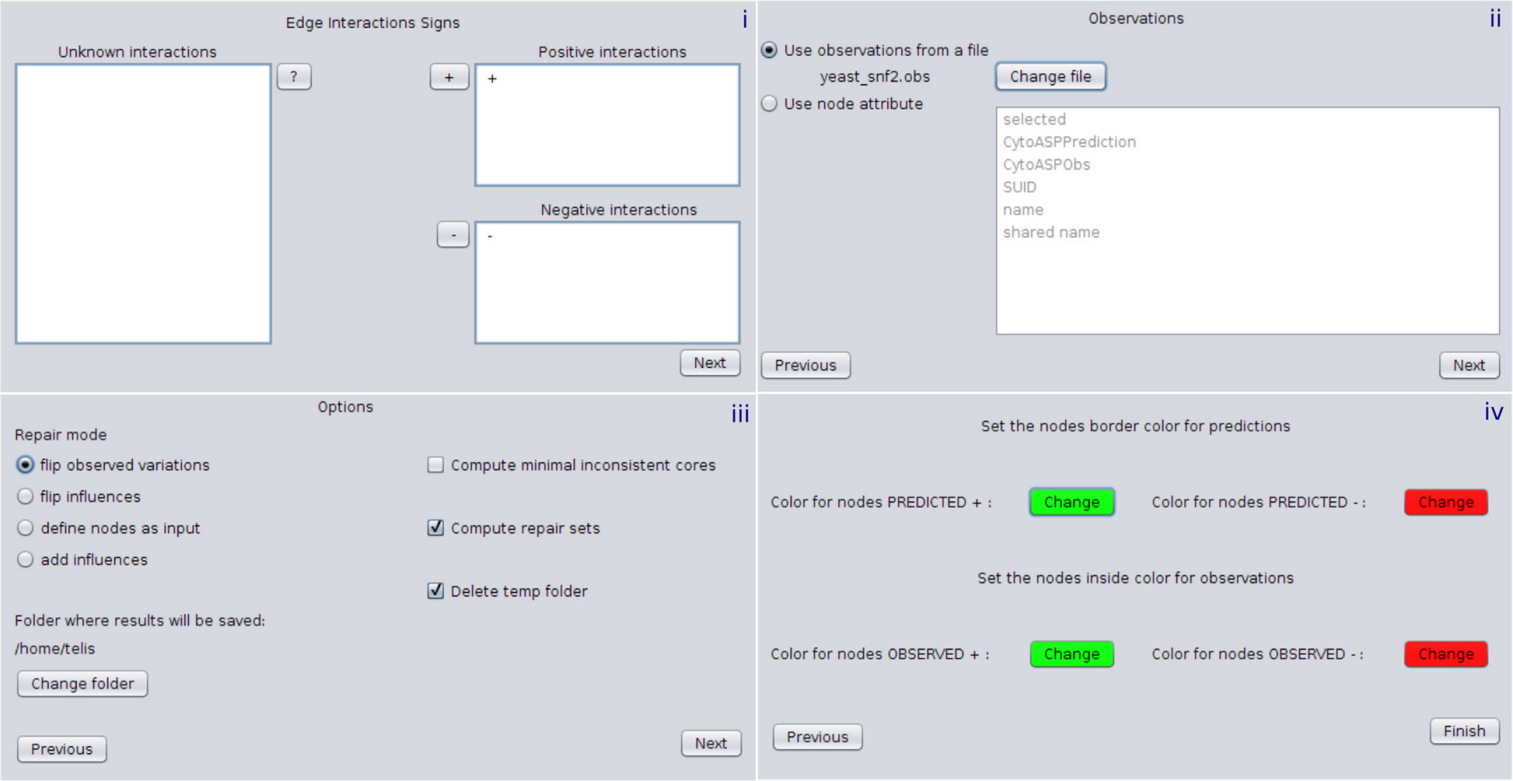
User interface of CytoASP. A typical workflow in CytoASP consists of four distinct steps: i. interaction assignment, ii. specifying observations iii. repair options and iv. visualisation options. After these options have been assigned the app proceeds to analyse the selected network and output the results

In a typical work session, the user loads a session in Cytoscape (Fig. 1), namely a set of networks and associated tables, classifying interactions between: i) positive, ii) negative and iii) unknown. The experimental profile is then specified, by either defining node observations as attributes or loading them from a file. For the first option, we can create column observations in the node table, and fill it either with relative variations or with −/ +.

After nodes and edges are assigned, the user defines repair options: flip variations, flip influences, turn nodes into inputs (i.e. not having their variation defined by any predecessors) or add edges. In the next step, the colours for visualising the results from computations are chosen. Once all options are set, the app calls *ingranalyze* to perform the computations and suggest predictions.

CytoASP is packaged as standalone software including solver binaries, Python libraries and Python itself. Therefore, it runs with no dependencies on any system where the BioASP solvers can be compiled. It is currently offered for Linux 64-bit systems as standalone software, due to its dependency on Python and availability of the BioASP solvers. We are considering a future implementation (CytoASP 2.0) providing execution via a web service to offer platform independence.

## Results and discussion

### Case study

We demonstrate the application of CytoASP on the yeast transcription regulatory network provided in [8], a genetic network comprising sets of genes that interact through directed transcriptional regulation. The yeast regulatory network contains 935 genetic or biochemical regulations, all of which have been established experimentally, among 447 genes. On this network, gene expression data from whole-genome microarray analysis of SNF2 knock-out mutants from [9] is overlaid. It is noted that SNF2 is a catalytic subunit of the SWI/SNF chromatin remodelling complex, that controls transcription by perturbing the structure of nucleosomes.

Genes that exert a regulatory role encode dedicated transcription factors can bind to specific DNA control regions of regulated genes to activate or inhibit their transcription. Regulated genes may themselves act in a regulatory manner, in which case they participate in a causal pathway. This transcriptional regulatory network is represented as a graph where vertices are genes and directed edges denote activating or repressing effects on transcription.

**Fig. 2:**
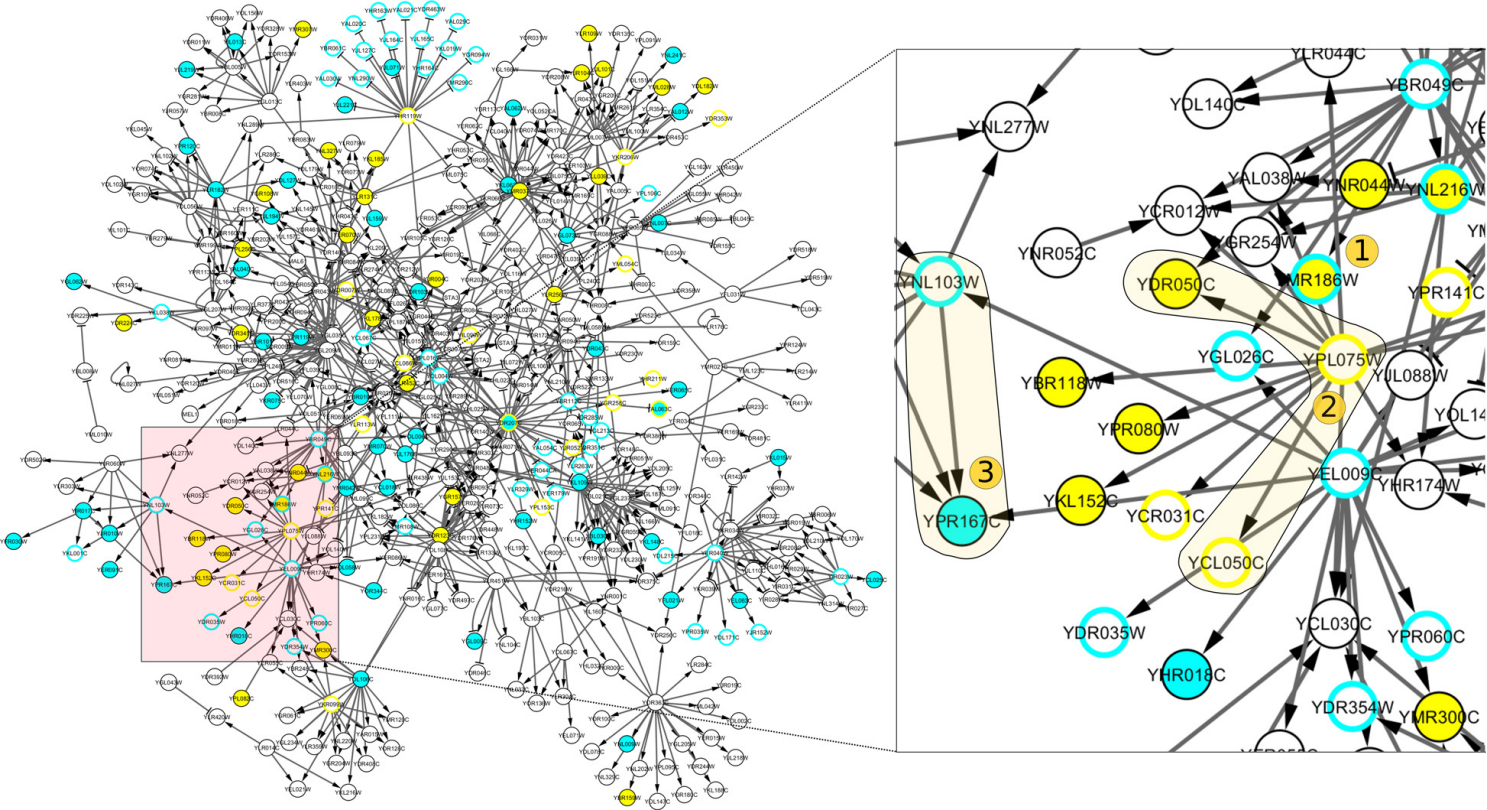
Network modelling in CytoASP. Overview of analysis done in network from [8], using transcriptional data from [9]. Detailed view of central part of the network, where one can spot regulations in observed and predicted nodes, as well as repairs common in all minimal repair sets. Predictions are presented as border-coloured nodes and observations as solid coloured, where cyan and yellow represent up and down regulation respectively. In the close-up view nodes are highlighted after post-processing the network exported from Cytoscape using the vector editing software Inkscape (https://inkscape.org/)

Comparing the yeast regulatory network with the genetic profile of SNF2, we found the data to be inconsistent with the network. Multiple repair modes, as outlined above, can be employed in order to re-establish consistency. In our case, repairs are suggested through CytoASP by flipping observations (Fig. 2). The network can be repaired with a minimal set of 11 operations, has 48 minimal repair sets and 71 predictions that hold under all repair sets. These results can be evaluated experimentally or through expert knowledge. CytoASP presents these repair suggestions through an output window screen, in addition to saving the corresponding files for predictions and repairs. Predictions are presented as border-coloured nodes (Fig. 2) and observations as solid coloured nodes, where cyan and yellow represent up and down regulation respectively. These colours can be customised by the user.

Such visualisation capability facilitates spotting repairs that hold under all repair sets, since these are visualised as coloured nodes with alternating border colour. As an example, consider node (1) HSC82 (YMR186W) (Fig. 2). This gene was observed as up-regulated in the microarray dataset however, as consistency checks have indicated that it is down-regulated in *all* repair sets, it is highly likely that gene expression measurement may be inaccurate and further scrutiny can be planned for verification. It is noted that similar visualisation is possible if flipping influences (edges) or defining nodes as input are selected as a repair options.

This approach also provides predictions for unknown expression, based on the transcriptomics data and underlying network. Consider e.g. nodes YCL050C, YPL075W, YDR050C (2) in Fig. 2. Expression of APA1 gene (YCL050C) is regulated by the carbon source and requires the protein GCR1 (YPL075W) [10]. GCR1 mutations substantially decrease the expression of glycolytic genes, such as TPI1 (YDR050C), compared to those in the wild-type strain [11]. The observed down-regulation of TPI1 allows to predict the subsequent downregulation of GCR1 and APA1 which is inline with observations from the literature.

Another example are MET4 (YNL103W) and MET16 (YPR167C) which are linked with a positive influence (3). Transcription of MET16 and other genes required for sulfate assimilation is activated in the absence of methionine. The up-regulation of MET16 allows us to predict the up-regulation of MET4. Mutation in the MET4 gene abolishes transcription of MET16, thus MET4 is essential for MET16 expression [12]. Therefore the model provides a plausible explanation for the up-regulation of MET16 via the MET4 transcription factor.

### Comparison with existing software

The COMA Cytoscape plugin [13] implements a method to facilitate consistency checks for gene expression studies given a gene regulatory network. This approach is tailored to transcriptional gene regulatory interactions and can identify local inconsistencies for genes that are controlled by a set of transcription factors. However, it is limited in determining specific consistent patterns of genes regulated by a number of transcription factors, it does not provide global predictions and it is not able to suggest repairs in the case of inconsistency.

Integer linear programming (ILP) has also been recently employed on interaction graphs to encode constraints on the qualitative behaviour of the nodes [14]. This approach determines topology-consistent explanation for responses of signalling nodes measured in a stimulus-response experiment and identifies the optimal subgraph of the given network topology which can best reflect measurements from a set of experimental scenarios. In contrast, our backend (*ingranalyze*) that uses BioASP does not focus on generating subgraphs, rather it suggests edge/node repairs on the given network structure.

Qualitative influence graph modelling is currently available in Cytoscape 2.x via the BioQuali Cytoscape app [15]. This approach uses ternary decision diagrams (TDDs) to answer the consistency problem. TDDs provide an efficient representation of all the possible solutions of a qualitative system of constraints, however computation time increases significantly for large networks. On the other hand, BioASP only calculates predictions under repair that hold true under all repair sets (i.e. not all possible), however it is capable of dealing problems of much larger size, rendering it ideal for modelling large scale regulatory networks.

A significant advantage of the ASP paradigm is its flexibility and extensibility. Defining a new variation value for a node, (e.g. such as the null-variation), can be done by adding new rules into the program, while in the TDD approach, this requires modification of a significant part of its implementation. BioASP also offers significant capability for repairs not found in BioQuali, such as calculation of all minimal repair sets either as nodes or edges, and identification of minimal inconsistent cores.

While CytoASP has the advantages of ASP due to its usage of BioASP as back-end, it also offers additional benefits. It has extended options for visualising repair options under any repair mode, and customising the visualisation of repairs. The app runs locally, but has no dependencies so the only requirement is an existing Cytoscape installation. Furthermore, it runs each network analysis in a separate process, allowing parallel processing of all selected networks, thus taking advantage of any multi-processing capabilities. A detailed comparison is available in Table 1.

**Table 1:** Feature comparison of CytoASP and BioQuali [15]

	CytoASP	BioQuali
*Execution Model*	Local	Remote (Web Service)
*Reasoning Engine*	ASP solvers (through ingranalyze & BioASP)	Decision Diagrams
*Parallel Processing*	Yes, multiple networks may be analysed simultaneously using all available CPU cores	No
*Visualisation Options*	Custom, user can select colors for visualisation	Fixed, colors are pre-selected
*Repair Options*	i) Flip observations, ii) Flip influences, iii) Define nodes as input, iv) Add influences	Neutralise influences
*Repair Visualisation*	Common repairs are visualised for all four repair modes	Neutralised influences
*Repair Sets*	Yes, all minimal repair sets for nodes / edges are calculated	No
*Minimal Inconsistent Cores*	Yes	No

In terms of memory requirements, CytoASP uses 64-bit versions of Cytoscape and ASP solvers and therefore it is practically limited by the theoretical limit of a 64-bit system architecture (16 exabytes). However, it is noted that memory requirement for a graph with *n* number of nodes increases by *O*(*n*^2^), therefore large graphs (e.g. >10^6^ nodes) may occupy a large amount of system memory. Since Cytoscape uses adjacency list as its internal representation format, sparse graphs will occupy less space in memory.

The performance of CytoASP depends on BioASP, since the latter is used as back-end to perform calculations and provide the consistency analysis, predictions and repairs. Modern ASP solvers are based on advanced Boolean constraint solving technology and thus provide highly efficient inference engines [7]. The computation of the network in this case study completes within seconds. The average runtime may vary, depending on feasibility of repair modes on consistent as well as inconsistent observations. A detailed analysis on repair and prediction times with respect to the corresponding repair operation is provided elsewhere [16]. The most demanding process in terms of computation time is the identification of MICs, which increases quadratically with respect to grounding times of gringo ASP solver, in line with ground instantiations for MIC encoding growing quadratically with the size of influence graphs. A detailed analysis in the run-times when calculating MICs is provided in [7].

## Conclusions

We have presented CytoASP, proposing a Cytoscape app for determining consistency and making predictions and repairs in influence graphs. The software takes advantage of the extensive BioASP functionality in manipulating influence graphs, allowing for the modelling of large scale regulatory networks and dealing with inconsistency with extensive repair capabilities.

CytoASP provides customised visualisation in predictions and repair options. Common repairs can be visualised in node and edge repair mode, in addition to determining all repair sets through BioASP. In addition, multiple networks may be selected and analysed simultaneously, taking advantage of parallel processing capabilities. This functionality may be easily incorporated into a network analysis pipeline, through Cytoscape, allowing further analysis and visualisation.

CytoASP implements seamless and user-friendly integration of modelling graphs through BioASP with visualisation and processing options in Cytoscape. Therefore, it greatly simplifies qualitative network modelling by enabling non-programming experts to apply logical reasoning in gene regulations networks, facilitating its use in relevant Systems Biology projects.

## Availability and requirements

- **Project name:** CytoASP
- **Project home page:**https://bitbucket.org/akittas/cytoasp
- **Operating system(s):** Linux 64-bit
- **Execution model:** Multi-threading implementation, allowing the analysis of multiple networks in parallel using all available CPU cores.
- **Programming language:** Java, Python
- **Other requirements:** Cytoscape 3.x
- **License:** MIT license

## Endnotes

^1^http://bioasp.github.io.

^2^https://github.com/bioasp/ingranalyze/.

^3^http://mobyle.genouest.org/cgi-bin/Mobyle/portal.py.
